# Vitamin D-Resistant Rickets Diagnostics and Treatment Challenges at Muhimbili National Hospital, Tanzania

**DOI:** 10.1155/2020/1547170

**Published:** 2020-01-28

**Authors:** Evance K. Godfrey, Fatima Mussa, Parvina Kazahura, Aika Shoo, Helga Naburi, Karim P. Manji

**Affiliations:** Department of Paediatrics and Child Health, Muhimbili University of Health and Allied Sciences (MUHAS), Dar es Salaam, Tanzania

## Abstract

*Introduction*. Rickets is softening of bones caused by defective mineralization of the cartilage in the epiphyseal growth plate, causing widening of the ends of long bones, growth retardation, and skeletal deformities in children. It can be classified into calciopenic and phosphopenic, each type with various subclasses. *Case Presentations*. We presented 2 cases, first of a 1 year and 4-month-old male, with a history of recurrent episodes of cough for 8 months and bowing of the legs 6 months prior to admission. Clinical and laboratory investigation was suggestive of vitamin D-dependent rickets, and he started vitamin D treatment with minimal response. The second case is of a 4 years and 7-month-old male who presented with developmental delay, poor weight gain, and recurrent chest infection and worsening of bone pain since 9 months of age. Laboratory investigation was suggestive of phosphopenic rickets, and he was started on treatment at 9 months of age with little improvement and at 4 years, he sustained multiple fractures and succumbed to severe respiratory tract infection and died at 4 years and 7 months of age.

**Conclusion:**

Rickets pose a diagnostic and treatment challenge in resource-limited countries, and clinical judgment and early initiation of treatment are important.

## 1. Introduction

Rickets is softening of bones caused by deficiency of vitamin D, calcium, or phosphate. This leads to a defective mineralization of the cartilage in the epiphyseal growth plate and subsequent widening of the ends of long bones, growth retardation, and skeletal deformities in children. Rickets is classified as calciopenic or phosphopenic according to the predominant mineral deficiency [1, 2].

Calciopenic rickets results from calcium deficiency commonly due to inadequate intake of vitamin D (essential for intestinal absorption of calcium) and rarely inadequate intake of calcium [2, 3]. Calciopenic rickets can also be caused by decreased activity of vitamin D, such as lack of conversion of 25(OH) D to the active metabolite 1, 25(OH) 2D (vitamin D-dependent rickets type 1 (VDDR-I), or resistance to the active metabolite (also known as vitamin D-dependent rickets type II (VDDR-II) due to mutations leading to dysfunction of the vitamin D receptor [2, 4–8].

Clinically, type 1 and 11 can be distinguished by presence of the following in type 11, alopecia, and elevated level of 1, 25(OH) 2D poor response to vitamin D, and hence needs treatment with infusion of calcium [1, 9, 10]. However, both types will be present with elevated ALP, low or normal calcium, low phosphorus, high-serum parathyroid hormone (PTH), and radiological changes like widening of the epiphyseal plate and cupping and fraying of the epiphyseal end of metaphysis [2].

Phosphopenic rickets is mainly due to renal phosphate wasting resulting from either the primary renal tubular defect or excess generation of phosphatonin, which inhibit phosphate reabsorption from renal tubules and also reduces vitamin D 1*α*-hydroxylation [10–13].

Phosphopenic rickets is usually hereditary, and X-linked dominant hypophosphatemic rickets accounts for more than 80% and remaining 20% are due to autosomal dominant hypophosphatemic rickets, autosomal recessive hypophosphatemic rickets, and hereditary hypophosphatemic rickets with hypercalciuria [11].

Rare cases of acquired phosphopenic rickets result from benign mesenchymal tumors that produce a factor that decreases the proximal renal tubular resorption of phosphate and also may be associated with renal loss of phosphate as a result of a generalized proximal tubular dysfunction such as Fanconi syndrome [11, 12].

Clinically, the patient will present with features of rickets, short stature bone pain, and dental abscess [10, 14, 15]. Laboratory findings include raised ALP, hypophosphatemia, hyperphosphaturia normal or mild decreased 1, 25 (OH) D, and normal PTH level [3, 10, 14, 15]. Treatment is palliative with a high dose of phosphorous and vitamin D [10, 14, 15].

The condition hypophosphatemic rickets was first described by Albright et al. in 1937, with a prevalence of 1 in 20,000 [14].

Calcipenic rickets can be distinguished from phosphopenic rickets by predominantly muscle weakness, involvement of all limbs, tetany, enamel hypoplasia, absence of dental abscess, low calcium, and marked elevated ALP parathyroid hormone. Treatment of calcipenic rickets is usually a high dose of vitamin D while phosphopenic rickets is by phosphorous and vitamin D [2, 11].

## 2. Case Presentation

We report a 1 year and 4-month-old African male, who was admitted to our hospital because of recurrent episode of cough for 8 months and bowing of the legs for 6 months prior to admission.

The recurrent episodes of dry cough were worse at night and early morning, and he also had episodes of wheezing but no bluish coloration of the mucous membrane. He attends the outpatient clinic monthly, and prior to his recent admission, he had been admitted twice in an interval of two months due cough and difficulty in breathing, which were successfully treated with nebulized salbutamol and antibiotics. There is no history of asthma or atopy on the family. During the course of his illness, he was noted to have inward bending of the lower limbs, which was more prominent when standing. He has no history of trauma, limb length discrepancy, or swelling. His anterior frontanelle closed at one year, and he had no history of hair loss or balding patches. He had his first tooth eruption at 7 months, and no report of pain or pus discharge from the teeth was noted. He attained early developmental milestones until the age of 7 months, and the delay was noted subsequently as he was unable to crawl, stand, and walk during the recent admission. He had no history of convulsions or use of anticonvulsant medications, hoarseness of voice, or history suggestive of malabsorption disorder, liver disease, or kidney disease. There is no history of consanguinity or similar disease in the family.

His mother received adequate prenatal care and had adequate sun exposure. She had no history of muscle or bone pain during pregnancy. She reports taking diet consisting of eggs and fish in here regular diet, and she had a good appetite and no reported illnesses or drug exposure apart from hematinics during the gestational period. Her pregnancy was uneventful, and she delivered by SVD at the gestational age of 38 weeks, a male baby weighing 3.1 kg and started breastfeeding on the same day. The baby was only exclusively breastfed for 2 months, formula milk was introduced at 3 months of age, and he was weaned at 6 months. Currently, he feeds on foods rich in vitamin D and had adequate intake in terms of quality and quantity and also had adequate sun exposure. Immunization is appropriate for his age, and his weight gain pattern is appropriate for his age.

On his recent admission, he was alert, had some palmar pallor, no angular stomatitis or chelitis, no craniotabes, and no dental caries. His respiratory rate was 53 cyles per minute with the lower chest in drawing, and oxygen saturation was 89% in room air. He had a dull percussion note on the left inframammary and infrascapular regions with vesicular breath sounds, and he had crackles on the left inframammary and infrascapular and axillary regions with a prolonged expiratory phase, but no obvious wheezes were heard.

**Figure 1:**
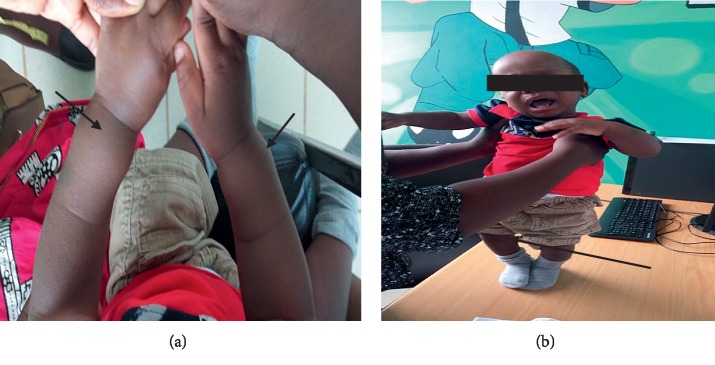
Enlarged wrist (a) and genu varus (b).

Musculoskeletal findings showed no rachitic rosary or Harrison's groove, but revealed skull bossing and a widened wrist and genu varus (Figure 1).

The other systems were essentially normal.

Blood investigations which were done on the initial visit are as shown in Tables 1 and 2.

He had elevated alkaline phosphatase (ALP) and serum parathyroid hormone with normal calcium, phosphorous, and 25D OH levels, consistent with stage 2 vitamin D-dependent rickets type 1 [2, 3].

Complete blood count results showed microcytic hypochromic anemia and leukocytosis with predominant neutrophilia consistent with sepsis [16].

The C-reactive protein (CRP) level was also raised suggesting pyogenic infection. Liver and renal functions were normal.

### 2.1. Images Limbs and X-Ray

**Figure 2:**
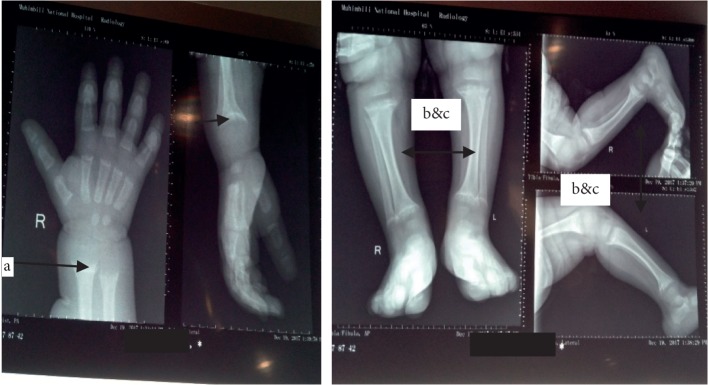
Radiological changes on the upper and lower limbs.

Radiographs of the distal radius and ulna bone (Figure 2) revealed (a) cupping/fraying in both the lower ends of the radius and ulna bone, (b) cortical thinning, epiphyseal widening, and X-ray of the proximal and distal tibia and fibula showed (c) bowing of legs and growth arrest lines. Chest X-ray showed opacification on the middle zone.

In view of the symptoms, physical examination, and investigation findings, we had a diagnosis of vitamin D-dependant rickets type 1, bronchial asthma, severe pneumonia, and moderate hypochromic microcytic anemia.

### 2.2. Treatment and Follow-Up

The patient was treated with salbutamol nebulization 2.5 mg 6 hrly for 48 hrs, then budenoside twice daily via a metered inhaler, then iv ceftriaxone 750 mg once daily for 3 days, and then was discharged home on syrup cefixime for 7 days. He was also started on calcitriol 0.25 *μ*gm once daily.

On subsequent outpatient follow-up clinic, his hemoglobin had returned to normal level and respiratory symptoms were improving. ALP was still high, and thus calcitriol was increased to a dose of 0.5 *μ*gm once daily and oral calcium supplements were added. At his 6 months of follow-up visit, he had a considerable improvement in his respiratory symptoms, ALP was still remarkably high 1100 IU/L, and other tests for monitoring of serum vitamin D, calcium, phosphorous, and PTH could not be done due to financial constrains. He still had persisted genu varus and was on vitamin D and calcium.

### 2.3. 2^nd^ Case

A 4-year-old male presented with developmental delay, poor weight gain, and recurrent chest infection for the past 3 years and worsening of bone pain since 9 months of age; he was delivered at the gestational age of 37 weeks, a male baby weighing 3.2 kg at birth and attained a maximum weight of 8.2 kg at 1 year. Thereafter, his weight was stagnant with a recent weight drop to 7 kg. With regard to his developmental milestone, his speech, social skills, and fine motor were appropriate for age, but he had delayed gross motor development as at age of 4 yrs he could not sit without support or stand which could be attributed to constant severe bone pain. His mother reported adequate nutrition intake and adequate sun exposure and she had optimal prenatal care with no history of bone pain. Tooth eruption was normal and no reports of abscess per oral cavity, alopecia, oliguria, or history suggesting malabsorption. He had a history of recurrent chest infection characterized by dry cough worse at night, which were being relieved by bronchodilators; he had no history of wheezes or bluish coloration of lips. His vaccination was up to date. He is the last born in a consanguineous Caucasian family of 5 children, with a history of sibling death at 1 year of age due to recurrent chest infections. Both parents were 38 years old at the time of consultation.

### 2.4. Physical Examination

On examination, he had normal hair distribution, his weight was 7 kg, and height was 83 cm making weight for length below –3SD and length for age below –3SD, consistent with severe wasting and stunting.

Has had widening of the wrists and genu varus on the limb, and he has no alopecia, dental abscess, or rachitic rosary.

Past investigations conducted at 9 months of age when he showed early signs revealing elevated alkaline phosphatase, low level of phosphorous calcium, and low serum vitamin D with serum parathyroid hormones within the normal range for age and sex are shown in Table 3.

These results showed elevated ALP low phosphorous, hypocalcemia, and normal PTH in keeping with phosphopenic rickets [3, 10].

### 2.5. Treatment and Follow-Up

He was kept on 5 *µ*gm per day of phosphorous and calcium supplements in the previous 6 months and yet control labs revealed low calcium, low phosphorous, and elevated urinary phosphorous levels and the parathyroid hormone levels were normal.

During a year of follow-up on a high dose of vitamin D and phosphorus, though there was no clinical or biochemical improvement, he still had abnormal results as shown in Table 3.

**Figure 3:**
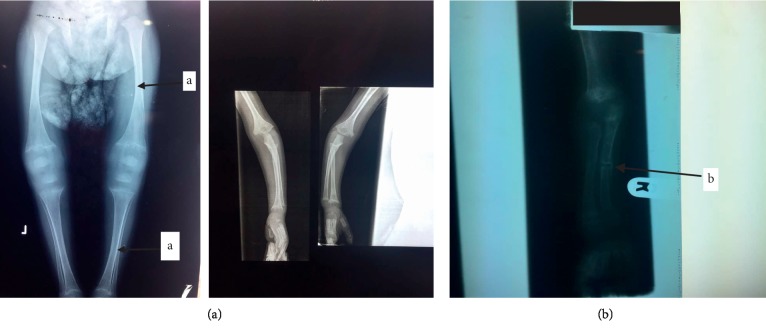
(a) Bowing of limbs with cortical thinning; (b) X-ray findings which were done at 4 years of age showed fracture of the midradius.

X-ray of the limbs repeated at the age of 4 years when he presented to our hospital showed a fracture of the middle radius, with significant cortical thinning (Figure 3).

The child died at 4 years and 7 months due to severe chest infection causing septicemia and before death was kept in mechanical ventilation and antibiotics, with no improvement, and the cause of death was respiratory failure.

## 3. Discussion

We reported two cases with similar clinical presentation where Case 1 had classical features of vitamin D-dependent rickets both in physical examination and laboratory and radiological findings with bowing of legs, elevated ALP, hyperparathyroidism, and normal calcium. This case also had recurrent chest infection and wheezes which can be explained by the fact that vitamin D is also important in both innate and adaptive immune response [17–19]. Based on several epidemiological studies, there has been reported increased episodes of bronchospams in children with vitamin D deficiency [20, 21]. Respiratory comorbidity can be severe enough to necessitate hospital admission as was a case report from Cape-Verde of a child who stayed in hospital for 9 months due to respiratory infections [22]. Another case report from Brazil reported prolonged hospitalization for 20 months during this time which necessitated mechanical ventilation and tracheostomy [23].

Our case also presented with anemia that can be explained by the fact vitamin D has a vital role in promoting erythropoietin by downregulating proinflammatory cytokines and activating the erythropoietin hormone itself [24–26]. Other typical features of rickets like craniotabes, dental deformities, and rachitic rosary and Harisson's groove were not present in this child. Laboratory findings were in keeping with vitamin D resistance due to high ALP, normal calcium due to PTH compensation, and elevation of PTH with normal phosphorous levels. This is due to the fact that hypocalcaemia is osteopenic [3]. Clinically, we were not able to distinguish between type 1 and 2 VDD rickets because there was no distinctive feature of alopecia or dental abscess which is a common feature of type 2 vitamin D-dependent rickets from several reported cases which were reviewed [9, 27–29]. Although this is not a diagnostic criteria, there are some reported cases of type 2 VDD rickets without alopecia [1, 7]. Other markers which would help distinguish between the two forms is the level of 1, 25D OH which is highly elevated for type 2 VDD rickets and reduced in type 1 [27]; unfortunately, we were unable to access this test.

Other important tests are genetic studies to identify mutation in the CYP27B1 gene encoding for the 1-alpha hydroxylase enzyme in which the gene is mapped on chromosome 12q14 [4].

Our patient responded well to other treatments including his respiratory complaints and anemia, but had suboptimal response to vitamin D as on the next follow-up ALP was still high and Ca was low. On reviewing the literature, it was suggested to escalate the dose of vitamin D for poor response to the initial dose [3, 6]. Poor response to a high dose of vitamin D could point towards type 2.

The 2^nd^ case we reported was of a clinical presentation of rickets with parental consanguinity and death of an older sibling with respiratory illness which suggests it could be an inherited condition. This child presented with poor weight gain and delayed motor and bone pain which are clinical characteristics of hypophosphatemic rickets; however, he has no dental manifestation as most of the published case reports reported either missing teeth or dental abscess [14, 15, 30]. Normal PTH and low phosphorous and 25 D OH and hyperphosphaturia are characteristic of phosphopenic rickets [3, 12]. Sadly, this patient had regular follow-up for 4 years with gradual deterioration of symptoms and of late had multiple fractures. There are reported cases of patients with fractures in this condition [27, 28]. These fractures pose challenge to the treatment as he already was on a high dose of vitamin D and phosphorous, but there was no clinical or biochemical response.

## 4. Conclusion

Rickets pose diagnostic and treatment challenges in resource-limited countries; clinical presentation alone cannot distinguish several types of rickets, and complete laboratory and genetic studies are important.

## Figures and Tables

**Table 1:** Results of blood investigation done on the first day of admission.

	ALP I (*µ*/l)	Calcium (mmol/l)	Phosphorous (mmol/l)	Magnesium (mmol/l)	Serum PTH (pg/ml)	25D OH (nmol/l)
Value	1180	2.25	0.72	0.79	182	100.75
Normal	100–644	2.1–2.6	0.7–1.3	0.7–1.2	10–55	100–250

**Table 2:** Complete blood count (CBC) results.

Hb	MCV	MCH	RDW	Plat	WBC	Neutr	Mon	LYM
9.06 gm/dl	67.4 fl	20.9 pg	24.1%	399 k/*µ*l	19.1 k/*µ*l	15.7 k*µ*/l	1.38 k/*µ*l	1.88 k/*µ*l

**Table 3:** Laboratory findings at 9 months and 1 year.

Serial number	Investigation	Normal level	At 9 months	1 year later
1	ALP	<281	1200 IU/L	1169
2	Calcium	2.2–2.6	2.19	2.02
3	Phosphorous	1.45–1.78	0.58	0.32
4	25D OH		66.75 nmol/l	
5	PTH	15–68.3	57.2	
6	Albumin			42 gm/dl
7	Urine phosphorous			32.21 mg/dl
8	Urine calcium	2.5–8 mmol/l		9.67 mg/dl
